# Chronic kidney disease and atrial fibrillation: A dangerous combination

**DOI:** 10.1371/journal.pone.0266046

**Published:** 2022-04-07

**Authors:** Gurbey Ocak, Meriem Khairoun, Othman Khairoun, Willem Jan W. Bos, Edouard L. Fu, Maarten J. Cramer, Jan Westerink, Marianne C. Verhaar, Frank L. Visseren, UCC-SMART study group

**Affiliations:** 1 Department of Internal Medicine, Sint Antonius Hospital, Nieuwegein, the Netherlands; 2 Department of Nephrology and Hypertension, University Medical Center Utrecht, Utrecht, the Netherlands; 3 Department of Clinical Epidemiology, Leiden University Medical Center, Leiden, the Netherlands; 4 Department of Internal Medicine, Leiden University Medical Center, Leiden, the Netherlands; 5 Department of Cardiology, University Medical Center Utrecht, Utrecht, the Netherlands; 6 Department of Vascular Medicine, University Medical Center Utrecht, Utrecht, the Netherlands; Kaohsiung Medical University Hospital, TAIWAN

## Abstract

**Background:**

Chronic kidney disease (CKD) and atrial fibrillation (AF) are both risk factors for bleeding, stroke and mortality. The aim of our study was to investigate the interaction between CKD and atrial fibrillation and outcomes.

**Methods:**

We included 12,394 subjects referred to the University Medical Center Utrecht (the Netherlands) from September 1996 to February 2018 for an out-patient visit (Utrecht Cardiovascular Cohort Second Manifestation of Arterial disease cohort). Hazard ratios (HRs) with 95% confidence intervals (CIs) for bleeding, ischemic stroke or mortality were calculated with Cox proportional hazard analyses. Presence of interaction between AF and CKD was examined by calculating the relative excess risk due to interaction (RERI), the attributable proportion (AP) due to interaction and the synergy index (S).

**Results:**

Of the 12,394 patients, 699 patients had AF, 2,752 patients had CKD and 325 patients had both AF and CKD. Patients with both CKD and AF had a 3.0-fold (95% CI 2.0–4.4) increased risk for bleeding, a 4.2-fold (95% CI 3.0–6.0) increased ischemic stroke risk and a 2.2-fold (95% CI 1.9–2.6) increased mortality risk after adjustment as compared with subjects without atrial fibrillation and CKD. We did not find interaction between AF and CKD for bleeding and mortality. However, we found interaction between AF and CKD for ischemic stroke risk (RERI 1.88 (95% CI 0.31–3.46), AP 0.45 (95% CI 0.17–0.72) and S 2.40 (95% CI 1.08–5.32)).

**Conclusion:**

AF and CKD are both associated with bleeding, ischemic stroke and mortality. There is a positive interaction between AF and CKD for ischemic stroke risk, but not for bleeding or mortality.

## Introduction

Recent studies showed that patients with chronic kidney disease have an increased risk of atrial fibrillation [1–3]. Activation of the renin- angiotensin-aldosterone system, increased inflammation, left atrial enlargement and diastolic dysfunction and myocardial fibrosis have been proposed as possible substrates for the development of atrial fibrillation in patient with chronic kidney disease [4]. Chronic kidney disease and atrial fibrillation are both associated with an increased risk of ischemic stroke [5–7], bleeding [7–9] and mortality [7, 10].

Although both atrial fibrillation and chronic kidney disease are associated with increased risks for ischemic stroke, bleeding and mortality, it is not known whether there is interaction between chronic kidney disease and atrial fibrillation in patients at high cardiovascular risk or patients with clinical manifest arterial disease. From a clinical point of view, it is important for clinicians to know whether ischemic stroke, bleeding and mortality risks are different for patients with chronic kidney disease and atrial fibrillation. Moreover, the clinical importance of atrial fibrillation in patients with chronic kidney disease is increasing over time, since incidences of both atrial fibrillation [11] and chronic kidney disease increase over time [12].

One of the main goals in patients with atrial fibrillation is to prevent ischemic strokes with anticoagulant therapy in patients with a high stroke risk. However, the use of antithrombotic drugs should be weighed against the increased risk of bleeding in patients with a chronic kidney disease [13]. In patients with chronic kidney disease and atrial fibrillation, there is uncertainty about the effectiveness and safety of anticoagulant therapy (vitamin K antagonists) with regard to stroke and bleeding risk [14, 15]. A very interesting group are patients at high cardiovascular risk or patients with clinical manifest arterial disease, since it is expected that both bleeding and stroke risks are high in this group, which makes therapy choices regarding antithrombotic drugs even more difficult than low risk groups.

The aim of our study was to evaluate the interaction between chronic kidney disease and atrial fibrillation on bleeding, ischemic stroke and mortality as outcomes in a large cohort.

## Methods

### Study population

The Utrecht Cardiovascular Cohort Second Manifestation of Arterial disease (UCC-SMART) cohort study, a single-center prospective cohort study, included patients aged 18–82 years, newly referred to the University Medical Center Utrecht (the Netherlands) for an out-patient visit with classical risk factors for arterial disease (hypertension, hyperlipidemia and diabetes mellitus) or with symptomatic arterial disease (coronary artery disease, cerebrovascular disease, peripheral arterial obstructive disease or abdominal aortic aneurysm). The UCC-SMART study was approved by the local Medical Ethics Committee of the University Medical Center Utrecht and written informed consent was obtained from all patients.

Patients underwent a standardized vascular screening program, including a health questionnaire and laboratory assessment and were contacted twice a year during the follow-up to fill in a questionnaire. Patients were included during the period September 1996 to February 2018, and then followed until March 2018. Patients were excluded when they had a terminal malignancy or when they were not sufficiently fluent in Dutch. A detailed description of the study was published previously [16]. The index date was the date of the first outpatient visit to the hospital after informed consent was obtained.

### Measurements

Creatinine was measured with enzymatic dry chemistry kits (Johnson and Johnson, New Brunswick, USA). Creatinine measurements were standardized to the reference method of isotope-dilution mass spectrometry. Albumin and creatinine concentrations were measured in a morning urine portion. Urinary creatinine was measured with dry chemistry kits (Johnson and Johnson) and albuminuria was determined with immunoturbidimetric assays (Boehringer-Mannheim, Mannheim, Germany). All assessments were performed at a single laboratory at baseline.

### Chronic kidney disease and atrial fibrillation

Glomerular filtration rate (GFR) was estimated by the Chronic Kidney Disease Epidemiology Collaboration (CKD-EPI) study equation [17]. The urine albumin-to-creatinine ratio was used to estimate albuminuria. A urine albumin-to-creatinine ratio less than 3 mg/mmol was considered normal and a urine albumin-to-creatinine ratio equal to or more than 3 mg/mmol was regarded as albuminuria [18]. Chronic kidney disease was staged according to the KDIGO guidelines [18]. Subjects with chronic kidney disease had either an eGFR <60 ml/min/1.73 m^2^ or had albuminuria (chronic kidney disease stages G1A2/3, G2A2/3, G3-5 with or without albuminuria) at baseline [18]. Data about the presence of atrial fibrillation at baseline were collected and confirmed by review of the medical history in electronic medical records and electrocardiograms of all patients.

### Outcomes: Bleeding, ischemic stroke and mortality

Bleeding was defined as a fatal or non-fatal hemorrhagic event after start of follow-up. This included any intracranial bleeding, fatal bleeding and any bleeding complication requiring hospitalization. Bleeding events were confirmed by evaluation of hospital discharge records and results of relevant laboratory and radiology examinations. Ischemic stroke was defined as having clinical features with increased impairment of ≥1 point on the modified Rankin Scale, without signs of hemorrhage on brain imaging (MRI or CT scan), and no other potential cause than ischemic stroke [19, 20]. Patients were followed until March 2018 for bleeding, ischemic stroke and mortality. Three members of the UCC-SMART committee independently audited all bleeding and ischemic stroke events on the basis of available information, and in the case of disagreement consensus was reached by consulting other members of the outcome committee.

### Data analyses

Baseline characteristics of the participants were compared between four groups: subjects without atrial fibrillation and without chronic kidney disease, subjects with atrial fibrillation and without chronic kidney, subjects without atrial fibrillation and with chronic kidney disease and subjects with atrial fibrillation and with chronic kidney disease. Continuous data were reported as medians with interquartile ranges. Person-years of follow-up were counted from the date of inclusion to the date of a first outcome event, death or the end of the study period (March 2018). Patients with incomplete follow-up were censored from the date of the last observation. Survival curves for ten-year mortality were determined with the Kaplan–Meier method stratified for the presence of atrial fibrillation and chronic kidney disease.

The presence of interaction was examined between atrial fibrillation and chronic kidney disease on bleeding, ischemic stroke and mortality. For this purpose, patients were categorized into four groups (patients without atrial fibrillation and without chronic kidney disease, patients with atrial fibrillation and without chronic kidney disease, patients without atrial fibrillation and with chronic kidney disease and patients with atrial fibrillation and with chronic kidney disease). Crude and adjusted HR with 95% CIs were calculated for each group using patients without atrial fibrillation and without chronic kidney disease as the reference category. HRs were adjusted for age, sex, body mass index, hypertension, stroke, myocardial infarction, peripheral arterial disease, heart failure, diabetes mellitus, use of anticoagulant drugs (vitamin K antagonists and direct oral anticoagulants), antiplatelet agents and hemoglobin levels. We investigated additive interaction by calculating the relative excess risk due to interaction (RERI), the attributable proportion (AP) due to interaction and the synergy index (S) [21]. In sensitivity analyses, we stratified the analyses for a CHA2DS2VASC score of zero or one (low-intermediate risk group) and CHA2DS2VASC group with a score of two or more (high-risk group) [22]. In addition, we stratified the analyses for users and non-users of antithrombotic agents (antiplatelet drugs or anticoagulant drugs). Analyses were done in SPSS statistical software version 23.0 (IBM SPSS Statistics) and R version 3.1.2.

## Results

### Baseline characteristics

**Fig 1:**
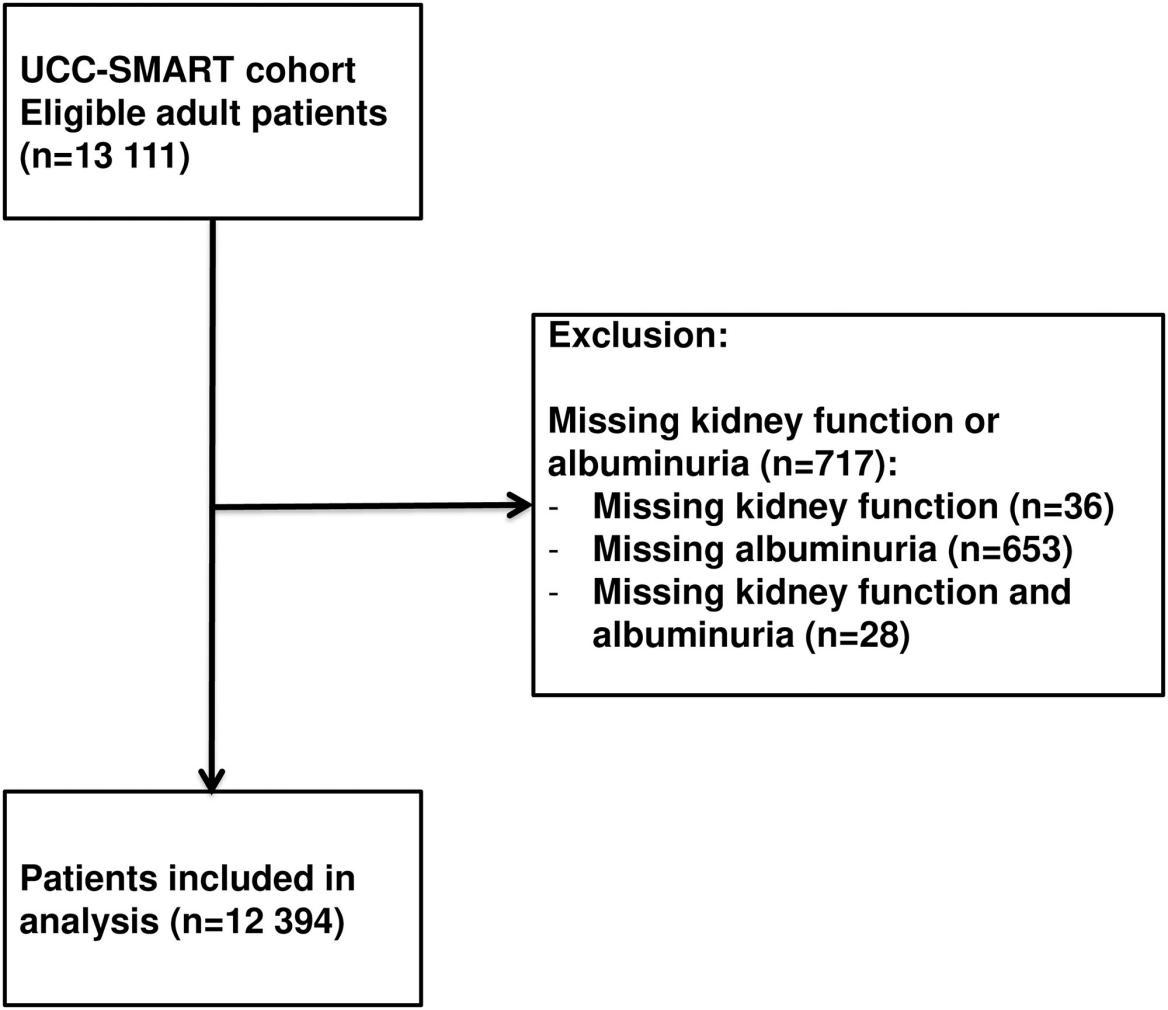
Study outline.

Of the 13,111 patients in our cohort, data on kidney function or albuminuria were absent in 717 patients leaving 12,394 subjects for the analyses. Fig 1 shows the study outline. Of the 12,394 subjects, 699 patients had atrial fibrillation and 2,752 had chronic kidney disease defined as eGFR<60 ml/min/1.73 m^2^ or urine albuminuria ≥3 mg/mmol creatinine. Patients with both atrial fibrillation and chronic kidney disease (N = 325) were older, were more often male, had a higher body mass index, had more often hypertension, history of stroke, myocardial infarction, peripheral arterial disease, heart failure, diabetes mellitus and used more often anticoagulant drugs than subjects without atrial fibrillation and chronic kidney disease (N = 9268) (Table 1).

**Table 1:** Baseline characteristics.

Atrial fibrillation	No	Yes	No	Yes
Chronic kidney disease	No	No	Yes	Yes
	N = 9,268	N = 374	N = 2,427	N = 325
Age (years) (IQR)	55 (46–63)	64 (58–70)	64 (55–70)	71 (65–75)
Sex, female (%)	3203 (35%)	88 (24%)	881 (36%)	91 (28%)
Body mass index (kg/m^2^) (IQR)	26.2 (23.8–28.9)	26.8 (24.5–29.9)	26.8 (24.3–29.8)	26.7 (24.2–29.6)
Waist circumference (cm) (IQR)	93 (85–101)	97 (90–106)	96 (88–105)	99 (90–107)
Hypertension (%)	4523 (49%)	224 (60%)	1778 (73%)	242 (74%)
Stroke (%)	1000 (11%)	65 (17%)	327 (13%)	51 (16%)
Myocardial infarction (%)	1810 (20%)	98 (26%)	553 (23%)	103 (32%)
Peripheral arterial disease (%)	936 (10%)	31 (8%)	374 (15%)	62 (19%)
Heart failure (%)	67 (1%)	8 (2%)	57 (2%)	26 (8%)
Diabetes mellitus (%)	1354 (15%)	66 (18%)	659 (27%)	102 (31%)
Antiplatelet drug use (%)	4975 (54%)	209 (56%)	1428 (59%)	159 (49%)
Anticoagulant use (%)	448 (5%)	170 (45%)	215 (9%)	157 (48%)
Statin use (%)	4842 (52%)	257 (69%)	1381 (57%)	193 (59%)
Total cholesterol (mmol/L)	4.9 (4.2–5.9)	4.6 (3.8–5.5)	4.9 (4.1–5.9)	4.9 (3.9–5.8)
HDL cholesterol (mmol/L)	1.2 (1.0–1.5)	1.2 (1.0–1.5)	1.2 (1.0–1.4)	1.2 (1.0–1.4)
LDL cholesterol (mmol/L)	2.9 (2.3–3.8)	2.6 (2.0–3.4)	2.8 (2.2–3.7)	2.8 (2.0–3.7)
Triglycerides (mmol/L) (IQR)	1.3 (1.0–2.0)	1.3 (1.0–1.3)	1.5 (1.1–2.3)	1.5 (1.1–2.1)
Hemoglobin (mmol/L) (IQR)	8.9 (8.4–9.4)	9.0 (8.3–9.4)	8.8 (8.1–9.3)	8.6 (8.0–9.3)
eGFR (ml/min/1.73 m^2^) (IQR)	85 (75–96)	80 (70–88)	58 (50–78)	55 (46–65)

**Notes**: Continuous variables are expressed as median with interquartile ranges (IQR).

### Outcome events: Bleeding, ischemic stroke and mortality

**Fig 2:**
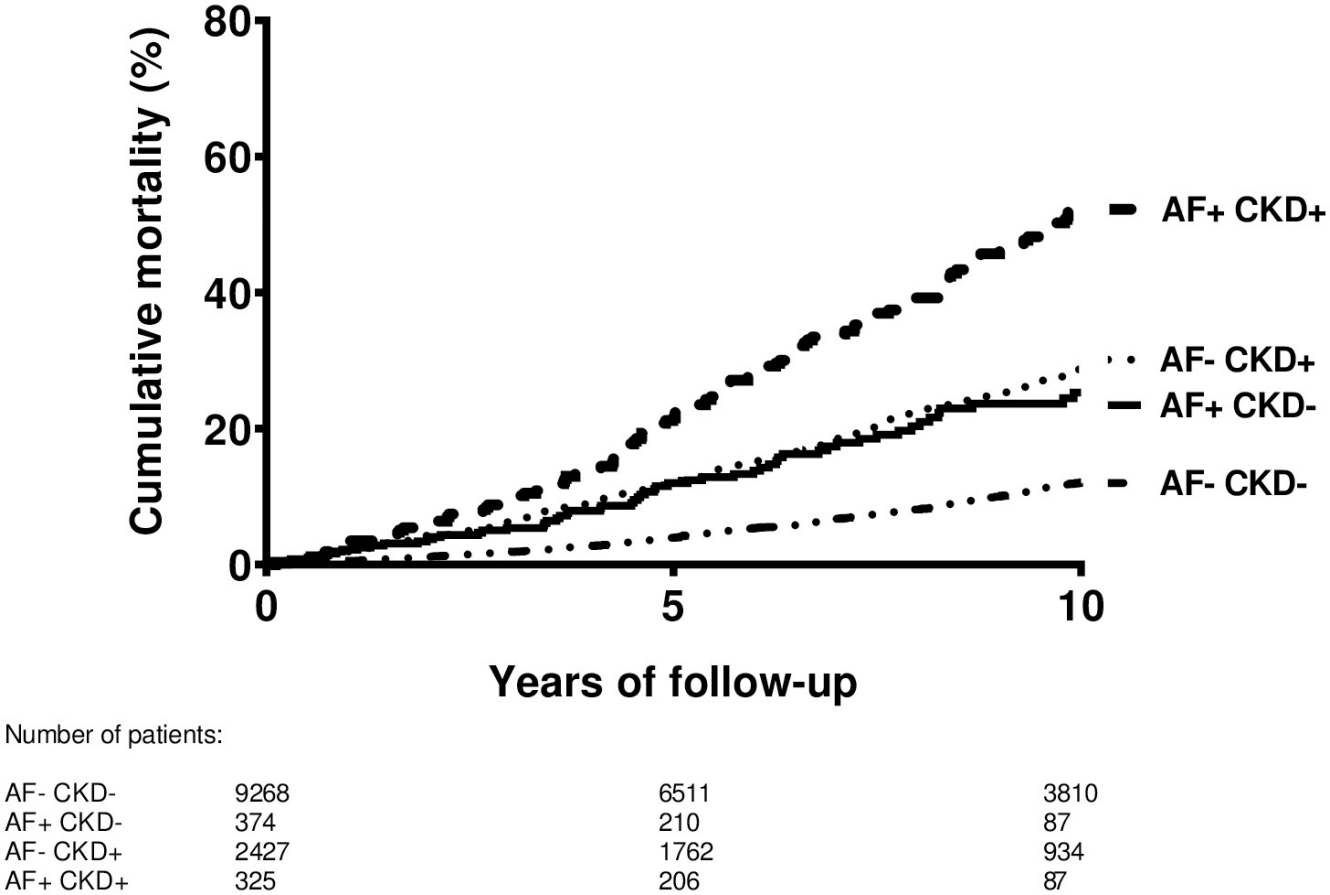
Kaplan-Meier survival curve for ten year mortality.

Of the 12,394 patients, 2203 died during follow-up time. The median follow-up time was 8.2 years (interquartile range 4.2 to 12.7 years) for the total group with a total follow-up time of 107 763 person-years. The median follow-up time was 8.4 years (interquartile range 4.2 to 13.0 years) for subjects without atrial fibrillation and chronic kidney disease, 5.8 years (interquartile range 2.9 to 9.8 years) for subjects with atrial fibrillation and without chronic kidney disease, 8.2 years (interquartile range 4.6 to 12.3 years) for subjects without atrial fibrillation and with chronic kidney disease and 6.4 years (interquartile range 3.7 to 10.4 years) for subjects with atrial fibrillation and chronic kidney disease. Fig 2 shows the Kaplan-Meier survival curve with ten year mortality as outcome. The cumulative mortality within ten years was 12% for subjects without atrial fibrillation and chronic kidney disease, 25% for subjects with atrial fibrillation and without chronic kidney disease, 29% for subjects without atrial fibrillation and with chronic kidney disease and 53% for subjects with atrial fibrillation and chronic kidney disease. Overall, 382 subjects developed a first bleeding event in the total cohort. Furthermore, 421 patients had a first ischemic stroke during the follow-up period.

### Combination of chronic kidney disease and atrial fibrillation on bleeding risk

Subjects without atrial fibrillation and chronic kidney disease had the lowest bleeding risk (incidence rate of 2.6 per 1,000 person-years) and patients with atrial fibrillation and chronic kidney disease had the highest bleeding risk (incidence rate of 17.2 per 1,000 person-years). The combination of atrial fibrillation and chronic kidney disease was associated with a 3.0-fold (95% CI 2.0–4.4) increased bleeding risk as compared with subjects without atrial fibrillation and chronic kidney disease (Table 2). There was no interaction between atrial fibrillation and chronic kidney disease and the risk of bleeding. The RERI was 0.62 (95% CI -0.75–1.99), the AP was 0.21 (95% CI -0.21–0.62) and the S was 1.45 (95% CI 0.62–3.43).

**Table 2:** Combination of atrial fibrillation and chronic kidney disease and risk of bleeding, ischemic stroke and mortality.

Atrial fibrillation	Chronic kidney disease		Number of events	IR	Crude HR (95%CI)	*Adjusted HR (95%CI)
**BLEEDING**						
No	No	N = 9,268	211	2.6	1 (ref)	1 (ref)
Yes	No	N = 374	18	7.7	2.7 (1.7–4.4)	1.9 (1.1–3.2)
No	Yes	N = 2,427	116	5.7	2.1 (1.7–2.7)	1.5 (1.2–1.9)
Yes	Yes	N = 325	37	17.2	6.1 (4.3–8.7)	3.0 (2.0–4.4)
**ISCHEMIC STROKE**						
No	No	N = 9,268	233	2.9	1 (ref)	1 (ref)
Yes	No	N = 374	19	8.0	2.7 (1.7–4.4)	1.8 (1.1–3.1)
No	Yes	N = 2,427	123	6.1	2.1 (1.7–2.6)	1.5 (1.2–1.9)
Yes	Yes	N = 325	46	21.3	7.3 (5.3–10.0)	4.2 (3.0–6.0)
**MORTALITY**						
No	No	N = 9,268	1,189	14.5	1 (ref)	1 (ref)
Yes	No	N = 374	75	30.7	2.5 (2.0–3.1)	1.4 (1.1–1.8)
No	Yes	N = 2,427	747	35.8	2.6 (2.4–2.8)	1.5 (1.3–1.6)
Yes	Yes	N = 325	192	81.7	6.5 (5.6–7.6)	2.2 (1.9–2.6)

**Abbreviations**: IR, incidence rate per 1000 person-years; HR, hazard ratio; CI, confidence interval.

Notes:

*Adjusted: Hazard ratio adjusted for age, sex, body mass index, hypertension, stroke, myocardial infarction, peripheral arterial disease, heart failure, diabetes mellitus, use of anticoagulant drugs (vitamin K antagonists and direct oral anticoagulants), antiplatelet agents and hemoglobin levels.

### Combination of chronic kidney disease and atrial fibrillation on ischemic stroke risk

Patients with both atrial fibrillation and chronic kidney disease had highly increased ischemic stroke risks (incidence rate of 21.3 per 1,000 person-years). The hazard ratio was 4.2 (95% CI 3.0–6.0) for the combination of atrial fibrillation and chronic kidney disease as compared with subjects without atrial fibrillation and chronic kidney disease (Table 2). We found an interaction between atrial fibrillation and chronic kidney disease. The RERI was 1.88 (95% CI 0.31–3.46), the AP was 0.45 (95% CI 0.17–0.72) and the S was 2.40 (95% CI 1.08–5.32).

### Combination of chronic kidney disease and atrial fibrillation on mortality risk

Mortality rates (81.7 per 1,000 person-years) were also highest for patients with both atrial fibrillation and chronic kidney disease leading to a 2.2-fold (95% CI 1.9–2.6) increased mortality risk as compared with the absence of atrial fibrillation and chronic kidney (Table 2). There was no interaction between atrial fibrillation and chronic kidney disease. The RERI was 0.34 (95% CI -0.12–0.81), the AP was 0.15 (95% CI -0.04–0.35) and the S was 1.39 (95% CI 0.86–2.25).

### Sensitivity analyses

As sensitivity analyses, we stratified for the CHA2DS2VASC score (Table 3). The combination of atrial fibrillation and chronic kidney disease as compared with subjects without atrial fibrillation and chronic kidney was associated with the highest risk of bleeding, ischemic stroke and mortality in both the CHA2DS2VASC group with a score of zero or one as the CHA2DS2VASC group with a score of two or more. Analyses stratified for antithrombotic therapy showed similar results (Table 4). The combination of atrial fibrillation and chronic kidney disease was associated with highly increased risks of bleeding, ischemic stroke and mortality in the both users and non-users of antithrombotic agents.

**Table 3:** Combination of atrial fibrillation and chronic kidney disease and risk of bleeding, ischemic stroke and mortality stratified for CHA2DS2VASC score.

				CHA2DS2VASC SCORE 0–1			CHA2DS2VASC SCORE ≥2
Atrial fibrillation	Chronic kidney disease		Number of events	*Adjusted HR (95%CI)		Number of events	*Adjusted HR (95%CI)
**BLEEDING**							
No	No	N = 4,424	82	1 (ref)	N = 4,844	129	1 (ref)
Yes	No	N = 108	4	1.4 (0.4–4.5)	N = 266	14	2.1 (1.2–3.7)
No	Yes	N = 511	8	0.6 (0.3–1.2)	N = 1,916	108	1.7 (1.3–2.3)
Yes	Yes	N = 25	2	3.0 (0.8–12.8)	N = 300	35	3.2 (2.1–4.9)
**ISCHEMIC STROKE**							
No	No	N = 4,424	62	1 (ref)	N = 4,844	171	1 (ref)
Yes	No	N = 108	2	0.6 (0.8–4.4)	N = 266	17	2.1 (1.2–3.6)
No	Yes	N = 511	15	1.7 (0.9–3.0)	N = 1,916	108	1.5 (1.1–1.9)
Yes	Yes	N = 25	7	14.0 (5.7–34.4)	N = 300	39	3.8 (2.6–5.6)
**MORTALITY**							
No	No	N = 4,424	362	1 (ref)	N = 4,844	827	1 (ref)
Yes	No	N = 108	20	2.0 (1.2–3.4)	N = 266	55	1.2 (0.9–1.6)
No	Yes	N = 511	89	1.5 (1.2–2.0)	N = 1,916	658	1.5 (1.3–1.6)
Yes	Yes	N = 25	13	4.6 (2.6–8.1)	N = 300	179	2.1 (1.8–2.5)

**Abbreviations**: HR, hazard ratio; CI, confidence interval.

Notes:

*Adjusted: Hazard ratio adjusted for age, sex, body mass index, hypertension, stroke, myocardial infarction, peripheral arterial disease, heart failure, diabetes mellitus, use of anticoagulant drugs (vitamin K antagonists and direct oral anticoagulants), antiplatelet agents and hemoglobin levels.

**Table 4:** Combination of atrial fibrillation and chronic kidney disease and risk of bleeding, ischemic stroke and mortality stratified for antithrombotic therapy.

				ANTITHROMBOTIC USERS			ANTITHROMBOTIC NON-USERS
Atrial fibrillation	Chronic kidney disease		Number of events	*Adjusted HR (95%CI)		Number of events	*Adjusted HR (95%CI)
**BLEEDING**							
No	No	N = 4,027	70	1 (ref)	N = 5,241	141	1 (ref)
Yes	No	N = 60	2	1.4 (0.4–5.9)	N = 314	16	1.9 (1.1–3.3)
No	Yes	N = 866	38	1.5 (1.0–2.4)	N = 1,561	78	1.5 (1.1–2.0)
Yes	Yes	N = 59	14	4.0 (2.1–7.8)	N = 266	23	2.6 (1.7–4.2)
**ISCHEMIC STROKE**							
No	No	N = 4,027	64	1 (ref)	N = 5,241	169	1 (ref)
Yes	No	N = 60	3	2.8 (0.9–9.2)	N = 314	16	1.6 (0.9–2.6)
No	Yes	N = 866	24	1.1 (0.7–1.8)	N = 1,561	99	1.7 (1.3–2.2)
Yes	Yes	N = 59	10	3.8 (1.9–7.9)	N = 266	36	3.9 (2.6–5.7)
**MORTALITY**							
No	No	N = 4,027	361	1 (ref)	N = 5,241	828	1 (ref)
Yes	No	N = 60	6	0.8 (0.4–1.8)	N = 314	69	1.5 (1.2–2.0)
No	Yes	N = 866	232	1.7 (1.4–2.0)	N = 1,561	515	1.4 (1.3–1.6)
Yes	Yes	N = 59	50	3.1 (2.2–4.2)	N = 266	142	2.1 (1.8–2.6)

**Abbreviations**: HR, hazard ratio; CI, confidence interval.

Notes:

*Adjusted: Hazard ratio adjusted for age, sex, body mass index, hypertension, stroke, myocardial infarction, peripheral arterial disease, heart failure, diabetes mellitus and hemoglobin levels.

## Discussion

In this large cohort study in patients at high cardiovascular risk, hazard ratios of bleeding, ischemic stroke and mortality were increased in the presence of both atrial fibrillation and chronic kidney disease as compared with subjects without atrial fibrillation and chronic kidney disease. We showed positive interaction between atrial fibrillation and chronic kidney disease in the risk of ischemic stroke, but not for bleeding and mortality.

Several previous studies investigated the association between stroke and atrial fibrillation in patients with chronic kidney disease [2, 23–25]. In a Swedish study, it was shown that patients with atrial fibrillation had a two-fold increased risk of ischemic stroke in patient with an eGFR below the 60 ml/min /1.73 m^2^ [2]. In a study from China, atrial fibrillation was associated with a two-fold increased risk of ischemic stroke in patients with chronic kidney disease [23]. Another study, that used diagnostic codes for atrial fibrillation and stroke event and did not take albuminuria into account, showed increased ischemic stroke rates with decreasing eGFR [25]. In the Global Anticoagulant Registry in the FIELD-Atrial Fibrillation study, chronic kidney disease as compared with no chronic kidney disease in patients with atrial fibrillation was associated with a one-year ischemic stroke risk. These studies were all in line with our finding of an increased ischemic stroke risk of patients with atrial fibrillation or chronic kidney disease. However, these studies did not investigate interaction between atrial fibrillation and chronic kidney disease [2, 23, 24], since there was no control group with patients without chronic kidney disease and without atrial fibrillation. In addition, our study included information about albuminuria, which was lacking in previous reports [2, 23–25], and outcome events were all confirmed and validated.

There could be several explanations for the increased risks of bleeding, ischemic stroke and mortality for the combination of atrial fibrillation and chronic kidney disease. Both chronic kidney disease and atrial fibrillation share common traditional risk factors such as an increased age, diabetes mellitus, hypertension and heart failure, which could explain the increased risks of bleeding, stroke and death for the combination of atrial fibrillation and chronic kidney disease [22]. Furthermore, the high prevalence of antithrombotic use in patients with chronic kidney disease and atrial fibrillation in combination with platelet dysfunction, especially in patients with severely decreased kidney function, could be an explanation for the association between the combination of atrial fibrillation and chronic kidney disease and bleeding [26, 27]. Finally, there is a recent debate about the effectiveness of vitamin K antagonist in subjects with severely impaired renal function and atrial fibrillation [15, 28, 29]. It could be that a decreased effectiveness of therapy in combination with arterial atherosclerosis and a prothrombotic state [30] in patient with chronic kidney leads to a synergic effect on ischemic stroke in the presence of atrial fibrillation. Although bleeding risks were extremely high for the combination of atrial fibrillation and chronic kidney disease, we did not find positive interaction for the combination of atrial fibrillation and chronic kidney disease on bleeding risk, probably due to a limited power. The RERI (> 0), AP (> 0) and S (> 1) were all increased in the interaction analyses, however it did not reach statistical significance.

There are several important notes for clinicians when treating patients with atrial fibrillation and chronic kidney disease. First of all, although we did not find interaction for bleeding and mortality, clinicians should be aware of the increased risks of bleeding and mortality in patients with a combination of atrial fibrillation and chronic kidney disease. Second, the finding that the combination of atrial fibrillation and chronic kidney disease highly increases stroke risk is important. Third, stroke risk scores and bleeding risk scores could have poorer performances in patients with chronic kidney disease as compared with patients without chronic kidney disease due to different risks than in the general population [31, 32]. Fourth, direct oral anticoagulants have been shown to be superior to vitamin K antagonists in patients with chronic kidney disease and an eGFR above 30 ml/min/1.73 m^2^ and are proposed as first line treatment in these patients if there is an indication for anticoagulation [33]. However, there are no trials on treatment of atrial fibrillation in patients with severely decreased kidney function and it is still unknown whether there is a preference for vitamin K antagonists, direct oral anticoagulant or conservative care without anticoagulant drugs because of a high bleeding risk.

The major strengths of the present study are the large number of participants with information on atrial fibrillation, kidney function and albuminuria with confirmed events of bleeding and ischemic stroke. This study has also several limitations. First, the study population comprised patients with or at an increased risk of cardiovascular diseases who were referred to a hospital. Therefore, there could have been selection bias due to the selection of patients with atrial fibrillation or chronic kidney disease who are more at risk for adverse outcomes than other patients. Furthermore, the results of the study cannot be generalized to all patients. Second, we had no information about the initiation or stopping of vitamin K antagonists and antiplatelet agents during follow-up. Third, we had no information about family history of cardiovascular diseases. This could have led to residual confounding, since family history of cardiovascular diseases could be a confounder in the association between cardiovascular outcomes and the combination of chronic kidney disease and atrial fibrillation. Fourth, we missed information about the duration of chronic kidney disease or atrial fibrillation. Therefore, we could not investigate the association between the duration of chronic kidney disease or atrial fibrillation and outcomes. Finally, we had no information about the development of atrial fibrillation or chronic kidney disease during follow-up. This has probably led to an underestimation of hazard ratios of bleeding, ischemic stroke and mortality in our study.

In conclusion, we showed that the combination of atrial fibrillation and chronic kidney disease was associated with highly increased risks of bleeding, ischemic stroke and mortality. Furthermore, we showed positive interaction between atrial fibrillation and chronic kidney disease in the risk of stroke, but not for bleeding and mortality.
